# Using wild relatives and related species to build climate resilience in *Brassica* crops

**DOI:** 10.1007/s00122-021-03793-3

**Published:** 2021-03-17

**Authors:** Daniela Quezada-Martinez, Charles P. Addo Nyarko, Sarah V. Schiessl, Annaliese S. Mason

**Affiliations:** 1 Plant Breeding Department, Justus Liebig University, 35392 Giessen, Germany; 2 Plant Breeding Department, The University of Bonn, Katzenburgweg 5, 53115 Bonn, Germany

## Abstract

Climate change will have major impacts on crop production: not just increasing drought and heat stress, but also increasing insect and disease loads and the chance of extreme weather events and further adverse conditions. Often, wild relatives show increased tolerances to biotic and abiotic stresses, due to reduced stringency of selection for yield and yield-related traits under optimum conditions. One possible strategy to improve resilience in our modern-day crop cultivars is to utilize wild relative germplasm in breeding, and attempt to introgress genetic factors contributing to greater environmental tolerances from these wild relatives into elite crop types. However, this approach can be difficult, as it relies on factors such as ease of hybridization and genetic distance between the source and target, crossover frequencies and distributions in the hybrid, and ability to select for desirable introgressions while minimizing linkage drag. In this review, we outline the possible effects that climate change may have on crop production, introduce the *Brassica* crop species and their wild relatives, and provide an index of useful traits that are known to be present in each of these species that may be exploitable through interspecific hybridization-based approaches. Subsequently, we outline how introgression breeding works, what factors affect the success of this approach, and how this approach can be optimized so as to increase the chance of recovering the desired introgression lines. Our review provides a working guide to the use of wild relatives and related crop germplasm to improve biotic and abiotic resistances in *Brassica* crop species.

## Climate change will result in a higher frequency of extreme weather events and increased pest and disease loads

Increasing concentrations of greenhouse gases resulting from industrial activity drive global warming via the greenhouse effect (IPCC 2014). Average temperatures are therefore rising globally, and are to date about 1 °C on average higher compared to pre-industrial levels (IPCC 2018), and about 1.5 °C higher over land (Shukla et al. 2019). Global temperatures will continue to rise a further 0.4–2.6 °C until 2050 depending on various climate protection policies (IPCC 2014). As a primary effect, rising temperatures increase the likelihood of heat waves (Shukla et al. 2019). Heat stress has negative impacts on plant growth due to its devastating influence on cell membranes and protein stability, and limits plant growth at all developmental stages, but particularly during flowering (Bita and Gerats 2013; Bailey-Serres et al. 2019). On top of direct effects, rising temperature can have two further adverse secondary effects on local climates: at warmer temperatures, the water holding capacity of the air increases about 7% per °C, which can lead to stronger single rain events and increase the likelihood of flooding (Trenberth 2011; Kodra et al. 2020). At the same time, rising transpiration can dry down soils more quickly and increase the likelihood of droughts (Trenberth 2011; Lu et al. 2019). Which outcome is more probable depends on season and geography. Central Europe, for example, can expect more rain in the winter season, but more drought in spring and early summer (Lu et al. 2019). Flooding leads to a loss of oxygen in the soil, which in turn leads to denitrification and ionic toxicity. Moreover, depending on how much of the plant is covered by water, flooding can also inhibit gas exchange and photosynthesis and therefore heavily impact plant metabolism (Sasidharan et al. 2018). Drought, on the other hand, leads to a loss of cell turgor, to which most crops react with closure of stomata (Iwaya-Inoue et al. 2018). This inhibits gas exchange and therefore leads to a loss in photosynthetic capacity (Chaves et al. 2009), with the production of reactive oxygen species as a negative side effect (Choudhury et al. 2017). Some farmers try to balance drought by increased irrigation when water resources are available, although this carries the risk of lowering ground water level and causing secondary salinification. The area of saline soils is also increasing, mostly due to unsuitable irrigation practices (Shukla et al. 2019), but also due to rising sea levels as a result of the ice shield melting and expansion of the oceans due to the warmer temperatures (Nerem et al. 2018; Cheng et al. 2020). Salinity negatively affects plant growth and survival, causing osmotic stress and ion toxicity (Chaves et al. 2009).

Finally, there are also tertiary effects of global warming. As climate zones start to shift (Shukla et al. 2019), insects and pests expand their climatic niche into higher latitudes and start spreading towards areas that were previously too cold for them (Suzuki et al. 2014). Moreover, increased abiotic stresses may weaken plant defense mechanisms against biotic stress (Suzuki et al. 2014).

The only putatively positive effect of rising industrial carbon dioxide levels is the fertilization effect via increased efficiency of the dark reaction of photosynthesis (Shukla et al. 2019). However, utilization of this effect depends on plant nitrogen and phosphorus availability (Sinclair et al. 2019) and is therefore mostly only expected in high-input farming. Moreover, the effect is expected to rapidly saturate due to the limited availability of RubisCO (Sinclair et al. 2019), such that additional rises in carbon dioxide are not going to increase growth further.

To summarize, the conditions for plant production are worsening quickly, and the available farm land is decreasing at the same time. Meanwhile, the global population is still rising, and we need to produce more food from less land and worse conditions than ever before. Therefore, crops need to be bred to produce more yield—we need to increase breeding gains. The major prerequisite for breeding gains is, however, genetic variation. In some crops, recent bottlenecks in breeding history have dramatically decreased genetic diversity within the gene pool, with *Brassica napus* (rapeseed) being a particular concern (Snowdon and Luy 2012). In this review, we introduce how *Brassica* wild relatives and the close relationships between crop species can be exploited to widen genetic diversity and improve resistances to biotic and abiotic stresses in this important group of crops, and outline potential methodology and considerations to using this approach in applied breeding programs.

## The use of wild relatives and related species for crop improvement in *Brassica*

The Brassicaceae, also referred to as the mustard family or the Cruciferae, are a family of flowering plants comprising 338 genera and 3709 species (Al-Shehbaz et al. 2006; Warwick et al. 2006). The Brassicaceae contains several species of research interest, including the model plant *Arabidopsis thaliana* (hereafter referred to as Arabidopsis), as well as crops such as *Raphanus sativus* (radish), *Eruca sativa* (rocket), *Sinapis alba* (mustard seed), and *Brassica napus* (rapeseed). Some species such as *Aurinia saxatilis* (basket-of-gold), *Iberis sempervirens* (candytuft), *Matthiola incana* (stocks), *Erysimum cheiri* (wallflowers) and *Lunaria annua* (honesty) from this family are cultivated as ornamentals. The Brassiceae tribe is one of the 49 tribes in the Brassicaceae family, and is a group containing a number of phylogenetic lineages originating from a single clade. The Brassiceae contains species of various ploidy levels, with chromosome numbers for 80% of the species in this tribe ranging from *n* = 6 to *n* = 75 (Warwick and Anderson 1993). The genus *Brassica*, in the Brassiceae, is made up of 37 species and is the most agronomically significant genus in the Brassicaceae tribe, and has undergone extensive domestication (Gomez-Campo 1980). This genera includes mainly herbaceous plants believed to have originated from the Mediterranean region, and modern adapted cultivars have a global distribution as cultivated vegetables and oilseed crop plants (Fahey 2003). *Brassica* crops are commonly consumed as leafy (pak choy, kale), stem (wasabi) and root (turnip, swede, rutabaga) type vegetables, spice crops (black or brown mustard), cooking oil (rapeseed) and feed for livestock. Next in agronomic significance from the mustard family are *Raphanus* and *Sinapis*, which are also useful as edible roots (radish) and condiments (white mustard seeds) respectively (Rakow 2004). Owing to their closeness as members of the same Brassicaceae family, *Brassica* species benefit from the numerous molecular genetics and genomic tools available to Arabidopsis (Snowdon 2007; Mason and Snowdon 2016). The close relationship between species of the *Brassica* genus combined with the ample wild relatives and minor crop species in the wider Brassicaceae tribe make it an interesting model for examining interspecific hybridization for crop improvement (Katche et al. 2019).

The Triangle of U, developed by Korean cytogeneticist Nagaharu U (U 1935), shows the evolutionary and chromosomal relationships between the A, B and C genomes of the diploid species *B. rapa* (AA, 2*n* = 20; turnip rape, turnip, Chinese cabbage, Pak choi), *B. nigra* (BB, 2*n* = 16; black mustard) and *B. oleracea* (CC, 2*n* = 18; cabbage, cauliflower, broccoli, kale, kohlrabi, Brussels sprouts), and their allotetraploids *B. carinata* (AABB, 2*n* = 34; Abyssinian or Ethiopian mustard), *B. napus* (AACC, 2*n* = 38; oilseed rape, spring rape, swede) and *B. juncea* (BBCC, 2*n* = 36; Indian or brown mustard) which were generated through spontaneous interspecific hybridization events between the diploid species. *Brassica napus* is a relatively young crop (< 10 000 years old) which originated from the spontaneous hybridization between turnip rape (*Brassica rapa*; AA, 2*n* = 20) and cabbage/kale (*Brassica oleracea*; CC, 2*n* = 18) (Chalhoub et al. 2014). Wild types of *B*. *nigra* have been found in parts of Europe, Asia and North Africa (Oduor et al. 2015). As reviewed by Rakow (2004), *B. nigra* (n = 8, B genome) was initially identified as a weed in cultivated fields in the Mediterranean region, and is commonly seen on road sides and fields near Tangiers, Morocco and under semi-cultivated conditions in Rhodes, Crete, Sicily, Turkey and Ethiopia (Vaughan 1977; Tsunoda 1980). *Brassica rapa* (*n* = 10, A genome) originates from the highlands near the Mediterranean sea from where it migrated northward into Scandinavia and westward into eastern Europe and Germany (Nishi 1980). According to various authors, *Brassica oleracea* (*n* = 9, C genome), (characterized with distinct phenotypes (Snogerup 1980)), is believed to be a seaside plant of northern European or Mediterranean origin. Wild *B. oleracea* varieties still exist on maritime cliffs and continue to grow along the coasts of northern Spain, western France, southern and southwestern Britain (Vaughan 1977; Fahey 2003). *Brassica carinata* has been cultivated in Ethiopia and neighbouring territories from ancient times, while many researchers agree that *B. juncea* is a plant of Asiatic origin, with Asia as a centre of major diversity (Chen et al. 2013).

Rapeseed, oilseed rape or canola (Canadian Oil Low Acid) is the third most important oilseed crop in the world. Oilseed rape generally refers to any member of the *Brassica* genus which is grown for edible oil (normally *B. napus*, *B. rapa* and *B. juncea*), while rapeseed technically refers just to *B. napus*. Rapeseed attained economic importance as a source of edible vegetable oil after intensive breeding programs that led to the production of lines with low erucic acid (< 2% in the oil), low glucosinolate content (< 30 mg/g in the meal) and increased yields. All these breeding efforts and intensive selection for agricultural purposes have led to the generation of elite varieties with low genetic diversity compared to the wider gene pools (Snowdon and Luy 2012). *Brassica napus,* via human-assisted migration, went from Europe (where it first originated) to other parts of the world because of its usefulness as a high yielding *Brassica* crop with high seed quality (Zou et al. 2010). Winter rapeseed first spread to Russia, then to Japan and later on to China, while spring rapeseed reached China via Canada (Wu et al. 2019). Presently, almost 60% of the total global rapeseed production is from Canada, China and India (www.fao.org/faostat/November 2018), with the EU and Australia as other major rapeseed producers. In addition to serving as a good source of edible vegetable oil, rapeseed is also a valuable animal feed ingredient for ruminants and monogastric farm animals, used in producing industrial compounds like lubricants and surfactants and also as a raw material for biofuels in diesel cars and tractors, mostly in Germany and Europe (Allender and King 2010; Zou et al. 2010; Friedt et al. 2018). Qualities that make canola the preferred choice of oil by nutritionists and consumers around the world include its high content of poly-unsaturated linolenic acid (richness in omega-3, ca. 10%) and high content of oleic acid, ca. 60% (Iniguez-Luy and Federico 2011; Friedt et al. 2018). However, the balance of uses in the brassicas need to be maintained, as the value of the vegetable brassicas is outstripping *B*. *napus* globally, especially in light of rapeseed losses due to insects since the removal of important chemical controls by the European Union.

## Useful traits identified in *Brassica* crops and wild allies

Each of the six major cultivated *Brassica* species contains unique, potentially useful agronomic traits that can be utilized to improve elite cultivars or to increase the gene pool within a species. While each species is often strongly associated with a particular phenotype, e.g. such that *B. napus* is widely known as a high yielding oilseed crop (76 MT produced in 2007, FAOSTATS) and *B. oleracea* as a highly variable vegetable type (Cheng et al. 2016), many traits present in individual species can be transferred between these closely related species for crop improvement. In the year 2009, a compendium of known traits in *Brassica* and wild relatives was published (Warwick et al. 2009). Since then, many other genotypes carrying relevant traits for agronomic improvement have been found in different *Brassica* accessions.

### Disease resistance traits

Disease resistance has been broadly studied due to the major impact on crop production and yield. Resistance to a particular disease can be governed by a single gene (e.g. an “R-gene”) or by many genes with minor effects (quantitative resistance). Although many *Brassica* cultivars have been identified to carry particular disease resistances, pathogen evolution rapidly overcomes individual resistance sources or types under the high selection pressure of cropping production systems, such that the need for new resistance alleles is an ongoing process. Clubroot (CR) disease caused by many identified pathotypes of pathogen *Plasmodiophora brassicae* is prevalent around the world and greatly affects production in *Brassica* cultivars (Dixon 2009). Major resistance to CR has been found, for example, in *Brassica rapa* (Karling and Karling 1942; Piao et al. 2009; Zhang et al. 2015a). A large-scale screening for CR resistance against pathotype 3 carried out in a collection of 955 *Brassica* accessions (mostly *B. rapa*), revealed highly resistant accessions of *B. rapa* (17)*, B. nigra* (4), and *B. oleracea* (2) (Peng et al. 2014). Another screening test of 22 CR isolates against 386 *Brassica* accessions (between 63–65 accessions of each species *B. rapa, B. nigra, B. oleracea, B. napus, B. juncea,* and *B. carinata*) revealed that most resistance sources were present in *B. nigra,* with some in *B. oleracea*, *B. rapa* and *B. napus* (but none identified in *B. juncea* or *B. carinata*) (Fredua‐Agyeman et al. 2019). Resistance to CR and downy mildew (*Peronospora parasitica* subsp. *brassicae*) was also tested in 52 accessions of *B.* *oleracea* and revealed frequent resistance to powdery mildew, but only a few lines possessed CR resistance (Carlsson et al. 2004). Further studies have also found field-based resistance to downy mildew in several *B. oleracea* lines (Monot and Silué 2009).

*Sclerotinia* stem rot (SSR), caused by *Sclerotinia sclerotiorum,* is a fungal disease that can cause considerable yield losses, with up to 70% infection incidence in winter oilseed rape when the conditions are suitable (Koch et al. 2007). Resistance for this disease has been identified in *B. oleracea* (Mei et al. 2011, 2013) and *B. napus* (Taylor et al. 2015), contrary to the high susceptibility found in *B. juncea* (Li et al. 2009). Recently, *Sclerotinia* resistance governed by several loci found in a wild C-genome species (*B. incana*) was introduced into *B. napus* via an interspecific hexaploidy hybrid bridge method (Mei et al. 2015, 2020). Through pyramiding three major QTLs, the BC_1_F_8_ line gained approximately 35% resistance when compared to the *B. napus* parent (Mei et al. 2020). Another strong source of resistance to SSR was found in *Brassica fruticulosa* (Rana et al. 2017). Subsequently, this resistance was transferred into a susceptible *B. juncea* genotype, producing introgressed lines with increased resistance, with a reduced lesion size of up to 69%. From the introgressed material it was also possible to select euploid and high pollen fertility lines, making it an excellent source to be utilized in future breeding programs (Rana et al. 2017).

Blackleg or phoma stem canker (caused by *Leptosphaeria maculans*) mainly affects rapeseed grown in Canada, Europe and Australia (West et al. 2001). One of the ways to control this disease is by sowing resistant cultivars; hence the need to find new genetic resources is always an ongoing process. Resistance to blackleg was found in lines of *B. napus* (Delourme et al. 2006; Rimmer 2006; Light et al. 2011) and *B. rapa* subsp. *sylvestris* (Yu et al. 2005, 2008). To date, no resistance R gene against blackleg has been observed in the C *Brassica* genome, although some possible *in silico* candidates have recently been proposed (Ferdous et al. 2020). Other kinds of resistances that involve more than just one gene are known as quantitative disease resistances. This resistance type is associated to particular genomic region/s or quantitative trait loci (QTL) that contribute to a partial level of disease resistance, usually more complex to identify due to its nature, but in the long term, harder for the pathogen to overcome (Pilet-Nayel et al. 2017). Several blackleg resistance QTL have been identified in spring-type *Brassica napus* (Larkan et al. 2016) and in diversity sets of *Brassica napus* (Jestin et al. 2011; Rahman et al. 2016; Raman et al. 2016).

*Brassica oleracea* is the major host for black rot (*Xanthomonas campestris* pv. *campestris*) (Vicente et al. 2001). This disease can cause severe damage, affecting up to 50% of the crop (Singh et al. 2011). Several resistant lines have been found in *B. oleracea* (Lema et al. 2012; Saha et al. 2016; Ribeiro da Silva et al. 2020) and *B. rapa* (Lema et al. 2015)*.* In search of resistance in other subgenomes than the C, a single gene resistance locus was identified in *B. carinata,* located on linkage group B7 (Sharma et al. 2016). Later on, this resistance was introgressed into *B. oleracea* using embryo rescue (Sharma et al. 2017).

Resistance to white rust (WR) caused by the pathogen *Albugo candida* has been found in *B. juncea, B. napus, B. rapa* and *B. carinata* varieties (Panjabi-Massand et al. 2010; Awasthi et al. 2012). Quite recently, a *B. juncea* Chinese vegetable type mustard called Tumida was found to be resistant to WR, for which a responsible locus was located on linkage group A06 (Bhayana et al. 2020). Different *Brassica* genotypes and allies from diverse origins were tested against *Pseudocercosporella capsellae* (white leaf spot disease) in field and/or controlled conditions, and genotypes from *B. carinata, B. juncea, B. napus, B. oleracea* and *B. fruticulosa* shown to be highly resistant (Gunasinghe et al. 2014, 2017). By comparing resistant and susceptible lines derived from the three allotetraploid *Brassica* types, Gunasinghe et al. (2016) identified a resistant *B*. *carinata* line possessing stomata prone to closure to inhibit pathogen penetration. Also, a higher stomata density was observed in the susceptible lines.

### Insect resistance traits

Insects also are a big problem in *Brassica* crops and major yield losses and aesthetic damage can occur under their attack. The major pests attacking *Brassica* belong to the order of Lepidoptera, Hymenoptera, Diptera, Homoptera and Coleoptera (reviewed in (Ahuja et al. 2011)), many of them with the ability to move and migrate to infest their hosts. A very common oilseed rape pest is the pollen beetle (*Brassicogethes aeneus,* previously known as *Meligethes aenus*), that can cause more than 80% yield losses (Hansen 2004). Unfortunately, to date, no natural resistance has been found and the only way to protect the plants is through insecticide application or other integrated pest management strategies. Due to this, new resistant insects have emerged (Spitzer et al. 2020) and novel strategies are required to control the pest (reviewed in (Hervé and Cortesero 2016)). For other pests, such as Diamondback moth (*Plutella xylostella*), that can cause severe economic damage (Zalucki et al. 2012; Li et al. 2016), resistance has been observed in a single line of *B.* *oleracea* spp. *capitata* (Kim et al. 2013)*.*

In 2015, 432 different accessions of *B. oleracea* and allies were tested against cabbage whitefly (*Aleyrodes proletella* L.), out of which 48 showed a high degree of resistance (Pelgrom et al. 2015). In this study, the wild relatives *B. incana, B. montana* and *B. villosa* were shown to be very unappealing to the pest under early growth and development conditions. One possible explanation for the observed resistance in *B. incana* is the presence of trichomes (absent in the susceptible genotype).

The pest known as cabbage seedpod weevil (CSW; *Ceutorhynchus obstrictus*) severely affects oilseed rape, especially during the early flowering period (reviewed in (Dosdall 2009))*.* Resistance for this pest has been found in lines produced by the cross of *Sinapis alba* (resistant parent) and *B. napus* (susceptible parent) (Tansey et al. 2010; Lee et al. 2014). Resistance to another weevil pest, *Ceutorhynchus napi*, also known as rape stem weevil, was found in resynthesized *B. napus* lines (Schaefer-Koesterke et al. 2017). The resistance observed might be due to antixenosis (non-preference) given the extended size of the stem and also the lack of specific glucosinolate compounds (Schaefer-Koesterke et al. 2017).

Fully developed cabbage root fly (*Delia radicum* L.) infests its host by laying eggs on the ground, close to the plant, where the larva can live by feeding from the roots, therefore affecting plant development and eventually damaging yield loss (Hopkins et al. 1999). In a panel composed of diverse *Brassica* species, the antibiosis resistance (adverse effects on the pest) of these plants against cabbage root fly was studied (Shuhang et al. 2016). Here they found high levels of antibiosis in *B. spinenscens* and *B. fruticulosa* under greenhouse conditions*,* given by the observed fewer eclosed flies per egg and reduced fly dry weight (Shuhang et al. 2016). Other potential resistance candidates more readily crossable with *Brassica* crops are accessions found in *B. montana, B. macrocarpa, B. villosa, B. hilarionis* (Shuhang et al. 2016) and *B. rapa* (Santolamazza-Carbone et al. 2017)*.*

In a 2-year case study, *Eruca sativa* cv. T 27 followed by *B. carinata* cv. DLSC 2 were the least infested by aphids (*Lipaphis erysimi*) under normal conditions when compared to *B. juncea, B. rapa* and a hybrid *B. napus* (Kumar and Sangha 2017). Between the species studied, there were different chemical profiles present in the inflorescence that can explain over 94% of the amount of aphids present (Kumar and Sangha 2017). Screening for resistance to the moth *Mamestra brassicae* was carried out in 21 cabbage (*B. oleracea* var. *capitata*) varieties (Cartea et al. 2010). The two more resistant varieties had the compact head characteristic, a morphological trait that can also be involved in insect resistance (Carmona et al. 2011). Some of the insect pests affecting *Brassica* plants can work as carriers of other diseases like viruses. There are several viral infections described as affecting *Brassica* crops, especially cabbage types, including for example cauliflower mosaic virus (CaMV), turnip yellow mosaic virus (TyMV) and turnip mosaic virus (TuMV) (Raybould et al. 1999). A combination of TuMV and CaMV infection can affect up to 25% of the yield in *B. oleacea* var. *capitata*, mostly due to TuMV, as no significant effect was observed when CaMV was inoculated alone (Spence et al. 2007), and in current times most research has focused on identifying resistance for TuMV. Turnip mosaic virus (TuMV) infection in crucifer plants was initially described in 1921 (Schultz 1921), where the characteristic spotted pattern of a “mosaic like virus” was observed in *Brassica rapa.* This disease is mainly transmitted by aphids and non-exclusively infects *Brassica* genotypes (Walsh and Jenner 2002; Shattuck 2010), causing a reduction in fitness, reproduction and quality of the plant (Maskell et al. 1999). The utilization of insecticides against aphids to control the spread of TuMV is not very efficient; consequently the identification and utilization of naturally resistant *Brassica* varieties becomes the preferred option to control the disease in an environmentally friendly way (Walsh et al. 1999).

In one study, *B. juncea*, *B. oleracea, B. rapa, C. sativa* and *R. sativus* lines were tested against TuMV virus pathotype 8 (Nyalugwe et al. 2015): different *B. oleracea* and *R. sativus* lines showed consistently extreme resistance to the virus. The rest of the lines showed different responses to the infection although there was potential for resistance in each of the species tested (Nyalugwe et al. 2015). Also in this study, a dominant gene conferring systemic resistance in *B. juncea* was identified (*TuMV RESISTANCE IN BRASSICA JUNCEA 01*) (Nyalugwe et al. 2015, 2016). Extreme resistance to the TuMV pathotype 8 has been also observed in 18 *B. napus* and 14 *B. carinata* lines from different origins (Nyalugwe et al. 2014). A resistance to TuMV virus found in *Raphanus sativus* was identified and successfully transmitted via somatic fusion with *B. oleracea* var. *capitata*, *B. oleracea* var. *botrytis, B. oleracea* var. *capitata*, to 61, 83.6 and 33.2% of the hybrids produced, respectively (Scholze et al. 2010).

### Abiotic stress tolerances

Abiotic stress tolerances also vary across the *Brassica* species. Salt tolerance has been shown to be greater in the allopolyploids *B. juncea*, *B. napus* and *B. carinata* than in their diploid parents (Ashraf et al. 2001). Similar effects were observed when comparing salinity tolerance between various *Brassica* genotypes and ploidies (Kumar et al. 2009). In a different study, where tetraploid turnips (*B. rapa*) were compared to diploid progenitors, it was also shown that this increase in ploidy positively affects salinity tolerance (Meng et al. 2011). A wide diversity set of *B. napus* accessions (85 inbred lines) were tested for salt tolerance under hydroponic conditions (Yong et al. 2015). The results showed significant variation in shoot fresh weight and dry weight between the different accessions and, at the same time, there was no correlation between sodium ion accumulation in leaves and the salt tolerance index.

Screening of nine different *B. juncea* genotypes resulted in the discovery of one tolerant genotype (Varuna) among them (Hayat et al. 2011). In *B. juncea,* several other tolerances have been observed, such as heat stress (Wilson et al. 2014) and cadmium tolerance (Gill et al. 2011; Irfan et al. 2014)*.* Nevertheless, many of the results obtained for tolerance to heavy metals depend on the methods utilized to screen the tolerance (Hernández-Allica et al. 2008) and also not all of them are easily comparable due to these differences (reviewed in (Mourato et al. 2015)).

Polluted soil, water or air can be of great danger to human health. Fortunately, we can use plants to remove those contaminants, a term known as phytoremediation (reviewed in Salt et al. 1998). An excellent example of this is *B. juncea* var. *foliosa*, which has the potential to be used in phytoremediation in thorium (Th) contaminated soils due to its ability to tolerate this metal (Zhou et al. 2016). Under low concentrations of Th, *B. juncea* var. *foliosa* grew better, but under high concentrations plant metabolism and growth rates were affected. Some of the *Brassica* vegetable types, like *Brassica rapa* subsp. *pekinensis* (Chinese cabbage), also have the capacity to accumulate high amounts of heavy metals without any obvious symptoms, presenting a potential risk for human food contamination (Xiong and Wang 2005).

Lack of water during flowering can heavily impact the final yield production of plants. Thankfully, we can use the available germplasm of a species to investigate how well they are able to cope, and even recover if they were submitted to water stress. Phenotyping for drought stress tolerance in *B. napus* under simulated normal and osmotic stress conditions in a hydroponic system combined with GWAS revealed 16 stress-tolerant accessions and 16 SNP loci associated with osmotic stress response (Zhang et al. 2015b). When comparing single genotypes of *B. napus*, *B. rapa* and *B. juncea* under simulated drought stress (using PEG-6000), it was found that *B. juncea* was more drought tolerant than the other two species (Alam et al. 2014). A study of drought tolerance in *B. napus* pre- and post-flowering conditions found 3 and 4 different accessions tolerant to drought, respectively (Zhu et al. 2011). A closer characterization of drought tolerance mechanisms in *B.* *napus* revealed that individual strategies vary strongly between accessions, but common drought tolerance genes might exist (Schiessl et al. 2020).

### Other traits of agronomic interest

A number of other miscellaneous traits of agronomic importance are also present in various *Brassica* species. Cytoplasmic male sterility (CMS) is a widely utilized system to produce F_1_ hybrids in *Brassica* crops taking advantage of the high hybrid vigour observed in seed yield (Yamagishi and Bhat 2014). Several systems have been found in species like *B. juncea* (hau CMS, (Wan et al. 2008))*, B. napus* (nap and pol CMS, (Brown 1999))*, B. rapa* (YSMS-6 (Bhajan 2000); eru CMS (Peng et al. 2015)), *Raphanus sativus* (ogu CMS (Ogura 1968)), *B. oleracea* (Zhiyuan et al. 1995; Fang et al. 1997) and a system produced by the cross between *B. napus* and *B. carinata* (NCa (Wei et al. 2009)).

Leaves are very important organs, where processes like photosynthesis, respiration and transpiration take place, and also are the initial barrier against environmental conditions. Leaf composition can also act as a barrier against herbivore attack (Žnidarčič et al. 2008; Bohinc et al. 2014). In *B. juncea* leaf morphology was studied in 10 wild accessions (Huangfu et al. 2009): the different populations varied in leaf thickness, wax content, and leaf surface, among other morphological traits. Interestingly, some of the phenotypes analyzed also correlated with herbicide (glyphosate) resistance in the populations, especially leaf thickness, with an R^2^ of 0.72.

Pod shattering, from an evolutionary point of view, is a great mechanism for seed dispersal. Unfortunately, from an economical point of view, in *Brassica* oilseed crops it can cause great seed losses during harvesting, which under normal conditions can reach up to 2–5%, and when the conditions are less than optimal values over 20% or up to 50% can be obtained (Price et al. 1996). Pod shatter resistance is present naturally in *B.* *carinata*, *B. juncea* and *B. rapa* genotypes (Raman et al. 2014). On the other hand, the variation present in *B. napus* for pod shattering is more limited. For example, in a study where 229 *B. napus* accessions were investigated for silique shattering resistance, just two varieties were fully resistant (Wen 2008).

Novel traits may also be utilized to produce niche *Brassica* types for different purposes. For instance, *Brassica* species are characterized by the ubiquitous presence of glucosinolates, although the amount and composition of these compounds varies depending on the tissue or cultivar analyzed (Verkerk et al. 2010; Sun et al. 2019). Glucosinolates are secondary metabolites that have been associated in plants with insect resistance (Evivie et al. 2019), fungal resistance (Bednarek et al. 2009; Buxdorf et al. 2013), signalling molecules in the auxin pathway (Katz et al. 2015), its involvement in other biological processes like flowering time and stomatal closure (reviewed in (Barco and Clay 2019)) and even the possible contribution in preventing certain types of human cancer like lung, stomach and prostate when included in the diet (reviewed in (Traka and Mithen 2009)). Most commercial oilseed *Brassica* cultivars have been bred to contain low levels of glucosinolates, which is more desirable for edible oil. However, there is a niche for specific glucosinolate profiles that are desirable for other applications, for example in industrial oil production (Princen 1979).

Carotenoid content is another trait of interest that can potentially be manipulated and bred to produce edible plants with specific profiles, to fit human needs. In *B. oleracea,* diverse carotenoid composition was observed in a set of 30 different cultivars from various origins (Mageney et al. 2016). In *B. rapa* spp. *pekinensis*, a hybrid produced by the cross of two incompatible cultivars produces a hybrid with orange inner leaves (Yangjun et al. 2005). Interestingly, also in *B. rapa* cultivars, the production of other pigments (anthocyanin) has been associated with cold and freezing resistance (Ahmed et al. 2015).

## Hybridization for crop improvement in *Brassica*

Genetic diversity within and between species is a prerequisite for breeding and crop improvement. In order to improve yields, increase disease resistance and refine oil qualities to cater to various nutritional and industrial purposes, it is imperative to introduce new sources of genetic diversity into existing elite cultivars (Allender and King 2010). In the Brassicaceae, new variation can be generated by hybridization involving adapted cultivars, wild types and landraces or exotic germplasm such as different species (Friedt et al. 2018).

Interspecific hybridization is useful in the introgression of desirable traits from one species to another and there are different approaches for transferring traits through interspecific hybridization (Prakash et al. 2009; Mason and Chèvre 2016). The success of crosses between any two parents can be determined by observing their pollen germination, pollen tube growth, embryo development, and seed set (Bhat and Sarla 2004). Hybrid incompatibilities occur when hybrids are sterile, less fit or even non-viable compared to their progenitors: this serves as a reproductive isolation barrier which can lead to speciation (Coyne and Orr 2004). Within the *Brassica* genus, incompatibilities may occur between different species, cultivars or species with different ploidy levels (Nishiyama et al. 1991; FitzJohn et al. 2007). To date, a number of genes with diverse functions, including those involved in oxidative respiration, nuclear trafficking, DNA-binding, and plant defence have been linked to hybrid incompatibilities (Johnson 2010; Rieseberg and Blackman 2010), but the underlying genetic and molecular mechanisms are not yet fully understood (Vaid and Laitinen 2019).

Other mechanisms that prompt hybrid incompatibility include conflicts resulting from the unequal parental contribution to the formation of hybrid or developing seed (Carputo et al. 2003; Johnson 2010; Köhler et al. 2010). This is often seen in the different phenotypes or success rate obtained when reciprocal crosses are made. In crosses between *Brassica* species, the choice of maternal species has a big effect on the success of the cross (FitzJohn et al. 2007; Chen et al. 2011). Another well-known example is the cytoplasmic male sterility (CMS) which is a maternally inherited trait characterized by the inability of a plant to produce functional pollen (Eckardt 2006). Hybrid chlorophyll deficiency causes white-coloured cotyledons and this has been identified to occur as a result of incompatibility between the plastid genome and the nuclear genome (Ureshino et al. 1999; Okamoto and Ureshino 2015).

Hybrid necrosis or death of young seedlings is another form of post-zygotic incompatibility which is associated with complexities in gene interaction (Potts and Dungey 2004; Okamoto and Ureshino 2015). In Arabidopsis for instance, it has been revealed that conflict between two gene variants or loci *(DANGEROUS MIX 1* (*DM1*) and *DANGEROUS MIX 2* (*DM2*)) may trigger defence reactions which can be detected phenotypically in hybrids as necrotic lesions on leaves and a decline in growth and fertility (Bomblies and Weigel 2007; Chae et al. 2014). *ACCELERATED* *CELL* *DEATH 6* (*ACD6*) is another gene that causes hybrid necrosis when its allele variants interact leading to the activation of pathogen-recognition receptors and trigger autoimmune response to pathogens in first generation hybrids of *A*. *thaliana* (Todesco et al. 2014; Tateda et al. 2015; Świadek et al. 2017).

Even after successful pollen germination and fertilization, the abnormal growth of the endosperm can interfere with normal seed development (Haig and Westoby 1991; Lafon-Placette and Köhler 2016). Similarly, in *Brassica* species, interspecific hybridization does not always lead to the production of mature seeds, as a result of irregularities in endosperm development (Nishiyama et al. 1991). Failure of endosperm development in hybrids may occur as a result of unbalanced parental genome dosages or genomic imprinting (Köhler et al. 2010).

## Transferring useful traits from wild relatives to crop species: how does it work?

**Fig. 1:**
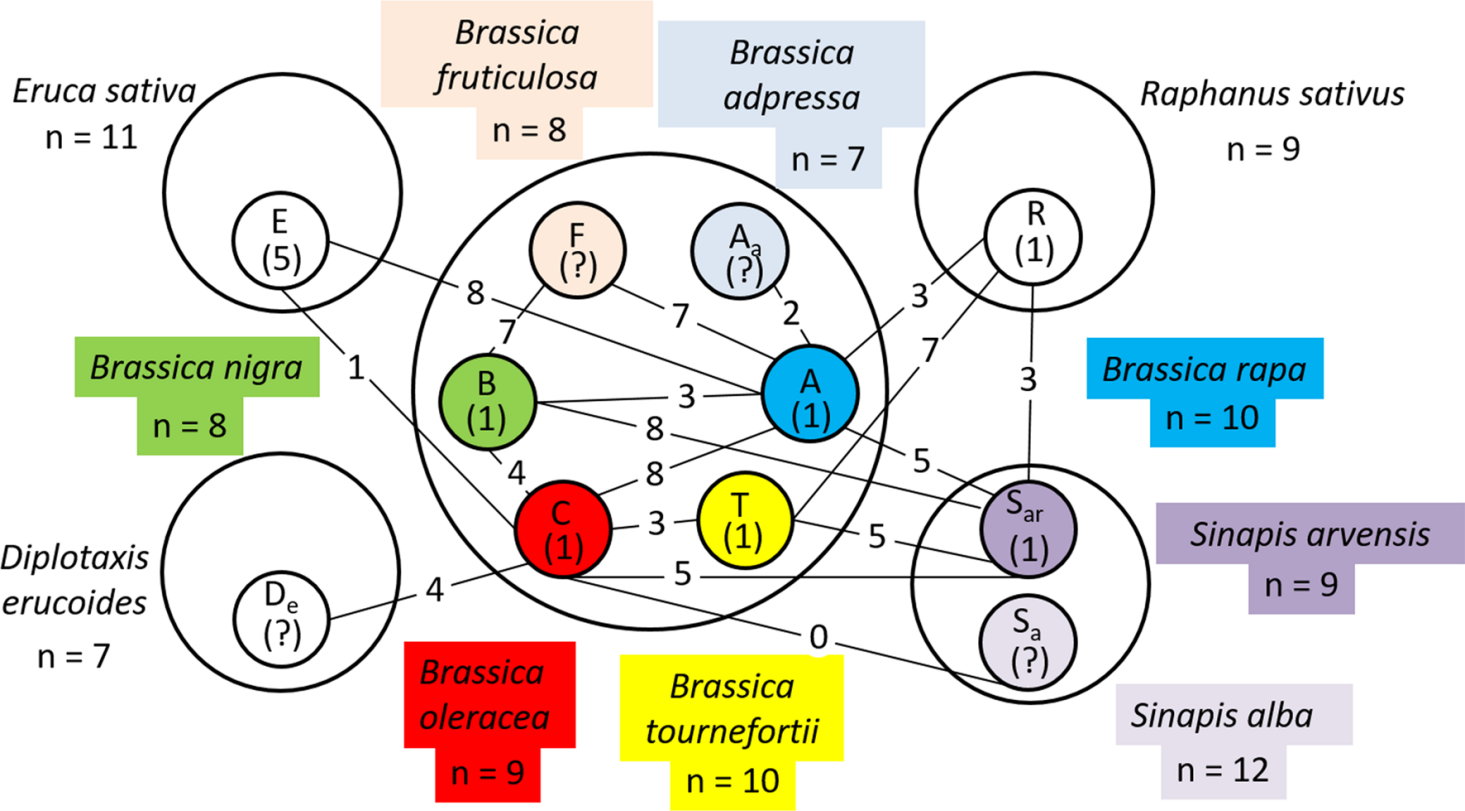
(adapted from Mizushima 1980): Genome interrelationships in *Brassica* and allied genera. Numbers in brackets represent the number of autosyndetic bivalents observed in haploids, while numbers on lines indicate the maximum number of bivalents observed in interspecific hybrids between the two species (necessary for transferring traits between genomes)

Although a major QTL *PrBn* (*Pairing regulator in* *B. napus*) and other minor QTL have been observed to affect non-homologous chromosome pairing frequencies in *Brassica napus* allohaploids (Jenczewski et al. 2003; Liu et al. 2006), *Brassica* species generally have weak, quantitative regulation of meiosis, which readily permits hybridization and introgressions to transfer useful traits between genomes (Fig. 1).

The first step in transferring useful traits from wild relatives to crops is to identify which wild relative germplasm carries the trait of interest, and preferably also the genetic basis for this trait. Ideally, the target germplasm will be within the same species, and the trait will be carried by a single major gene locus. Unfortunately, this situation is rarely found. Firstly, many species are relatively inbred, lacking the genetic and trait diversity necessary for further specific improvements. In the *Brassica* genus, this is particularly true in major crop species *B. napus* (rapeseed), for which no “wild” forms exist (Dixon 2007), and in which (for example) little to no resistance to insect predation is thought to exist (Hervé 2018). Hence, it is often necessary to look outside this so-called primary germplasm pool for traits. Secondly, although some traits are often carried by major genes, such as resistance to blackleg/Phoma disease (Rimmer 2006; Leflon et al. 2007) or resistance to clubroot (Manzanares-Dauleux et al. 2000), most traits, including drought tolerance (Fletcher et al. 2015, 2016; Zhu et al. 2016), flowering time (Schiessl et al. 2014, 2015), and of course yield (Zhou et al. 2014; Luo et al. 2017), tend to be the product of multiple genes, genetic factors or gene networks.

**Fig. 2:**
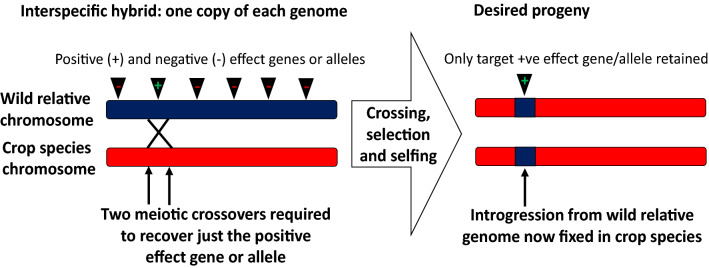
Meiotic crossovers in the interspecific hybrid are required between chromosomes belonging to the wild relative and the crop species (homoeologous crossovers) for production of introgression lines

The reason that it is better to have traits which are (a) present in closely-related species and (b) controlled by a single locus is because of the mechanisms by which we transfer traits from the wild to crop germplasm. The physical transfer of genetic material between two germplasm groups usually needs to occur via one or more crossovers between chromosomes in the hybrid which has been produced between them, which (usually) has 50% genetic material from each parent (Fig. 2).

While it is relatively easy to make hybrids within a species by hand-emasculation and pollination, this becomes much more difficult with increasing genetic distance between the wild relative and the crop (FitzJohn et al. 2007). As well, the subsequent chance of recovering recombination events is greatly reduced if there is little relationship between the two sets of chromosomes present in the hybrid, such that they rarely pair and recombine with each other (Mason and Chèvre 2016)(Fig. 1). If multiple genetic loci need to be transferred, even more crossovers need to form, and this further reduces the chance of recovering the desirable trait in segregating hybrid progeny (Mason and Chèvre 2016). Depending on the genomic location of the locus of interest, it may not even be possible to produce recombinants through conventional means, as crossovers are not evenly distributed across chromosomes (reviewed by (Choi and Henderson 2015)), and are actively suppressed in others, such as centromeres (Talbert and Henikoff 2010). Hence, some genomic regions are very unlikely to recombine during meiosis: when considering two target regions in a hybrid, the probability of a natural crossover forming between these two may be so low as to be effectively non-existent. Also, every transfer has the potential to introgress large blocks of undesirable genetic variation as well as the desirable genetic variation conferring the trait of interest (linkage drag), as normally, a large chromosomal segment will be introgressed from a single crossover. In the case of intraspecific crosses or crosses between species with very high genomic similarity this is not such a big problem: subsequent recombination events may occur through backcrossing to the crop parent, and thus reduce the size of the introgression block (Fig. 2). This eliminates undesirable genetic variation while retaining the locus of interest. However, further recombination events cannot be guaranteed in the case of wide crosses, which may mean that the resulting introgression region is large and carries a high number of undesirable genetic variants. This problem was classically encountered in *Brassica* breeding with the production of the restorer lines for the “Ogura” CMS system developed from radish wide hybrids (Pellan-Delourme and Renard 1988), and for which gamma ray induction of chromosome breakage was required to reduce introgression size (Primard-Brisset et al. 2005).

## Improving our chances of recovering introgressions of useful traits from relatives into crops to build climate resilience

So, what can we do to facilitate transfer of genetic loci and traits of interest from wild relatives into crops to build climate resilience? Good experimental planning and prior knowledge is key to improving success rates. Although in some cases very little is known about (a) genetic control of the target trait or phenotype in question, (b) ease of hybrid production, (c) frequency and distribution of crossovers in the interspecific hybrid or (d) chance of recovering successful introgressions, most of the time at least some of this information should already be known, and can be used to predict the amount of time and effort likely required to achieve this goal. Recent developments in genomics and bioinformatics techniques are predicted to help a lot in this respect (for review see (Zhang and Batley 2020)). However, there are also a number of specific methods or considerations that can be used to facilitate this process.

Hybrid generation is not always successful, especially across different ploidy levels. However, a number of crossing approaches can be used to facilitate trait transfer through interspecific hybridization in *Brassica* species and their relatives (Prakash et al. 2009; Mason and Chèvre 2016). Hybridization is generally more successful between species which share a genome, e.g. between a tetraploid and a diploid progenitor species, or between two tetraploids which share a progenitor ((Prakash et al. 2009); reviewed in Mason and Chèvre 2016). Hybridization between diploids and tetraploids that do not share a genome is also possible, and the resulting tri-genomic hybrids can be used as a bridge to introgress genetic diversity between species in further hybridization events, or can be induced by colchicine doubling to generate allohexaploids (Chen et al. 2011). While interspecific hybridization is very useful for hybrid speciation and crop improvement in the *Brassica* genus, hybridizations can also be made between genera. Hybridization involving the *Brassica* crop species is often successful using only hand-pollination methods (FitzJohn et al. 2007). However, hybridization can be facilitated by tissue culture techniques which “rescue” fertilized ovules or embryos before these are aborted by the maternal parent (reviewed by Sharma et al. 1996). For wider crosses somatic fusion may also be possible, where somatic cells (usually protoplasts) are directly induced to combine in tissue culture (reviewed by (Navrátilová 2004)), although this method frequently results in aneuploidy (loss or gain of individual chromosomes from a set) (Gaebelein and Mason 2018). A common example is the protoplast fusion of rapeseed and radish. This method was employed in generating the Ogura cytoplasmic male sterile *B. napus* system and was very beneficial in attaining double low restorer lines with a decrease in erucic acid and glucosinolate content (Pelletier et al. 1983; Primard-Brisset et al. 2005). Better success may also be achieved in some cases by chromosome doubling the parent species before hybridization is attempted (Frandsen 1947; Heyn 1977; Akbar 1990); this can also be achieved by various chemical treatments and methods (reviewed by (Dhooghe et al. 2011)). Other approaches used in tackling these incompatibilities include hot water treatments against pre-fertilization barriers (Prabha et al. 1982), early pollination of stigmas or stump pollination, in vitro pollination (reviewed in Katche et al. 2019), and artificially supplied nutrients and hormones against post-fertilization barriers (Sharma et al. 1996; Abel et al. 2005; Prakash et al. 2009).

Once a hybrid is produced, every meiosis in this hybrid (every pollen or ovule produced) has the potential to produce recombinant chromosomes (introgressions) between the source and target genomes. Meiotic recombination is an important aspect of breeding, as it ensures plant fertility and the generation of diversity through shuffling of genetic information. However, CO localization is uneven across the genome, with 80% of all COs occurring in about 25% of genomic regions in most plants (usually at the distal euchromatic regions) (Darrier et al. 2017). Obtaining the meiotic recombination required for crop improvement is also challenging in plants with low CO frequencies. Knowledge of how often recombination events occur in different types of hybrid is invaluable in knowing approximately how many progeny may need to be obtained in order to recover the desired introgression. Even better, knowledge about genome-wide distribution of recombination rates in hybrids of different types would allow predictions of success in introgression of particular genetic loci before experiments even start. However, COs can potentially be increased through several mechanisms, including the knockout of anti-crossover regulators such as FANCM, RECQ4 and FIGL1, or combining the knockout of anti-CO regulators with an increase in the dosage of ZMM protein HEI10, and through mutagenesis approaches (Blary and Jenczewski 2019). In *Brassica* allotriploid AAC hybrids, there is an increase in CO rates between the homologous A chromosomes (Leflon et al. 2010); better understanding and characterization of this effect may be helpful in applying this crossover boost to other hybrid types. In future, it may also be possible to manipulate the regulation of chromosome pairing between genomes in order to boost the frequency of crossovers and change their genomic locations (reviewed by Blary and Jenczewski 2019).

To facilitate introgressions in the absence of other information about recombination frequencies or crossover distributions, target genes should ideally be close to chromosome telomeres and in chromosomal regions with high homoeology (or homology if possible) between the two sets of chromosomes in the hybrid. In *B. juncea* × *B. carinata* BBAC hybrids, A-C pairing is extraordinarily frequent, with an average of 7 A-C allosyndetic pairs per meiosis (Mason et al. 2010), almost always between primary homoeologous regions (Mason et al. 2014). In the same hybrids, autosyndetic recombination events (A-A and C–C in the haploid genomes) occur at a rate of approximately one event per 2 meioses (Mason et al. 2010), but likely only between the largest blocks resulting from the ancestral genome triplication, involving up to a chromosome arm (Mason et al. 2014). In hybrids resulting from the cross *B. napus* × *B. nigra*, B-A/C allosyndesis is observed in 1/3 meioses in ABC triploid hybrids, and 1/6 meioses in AABBCC allohexaploid hybrids (Gaebelein et al. 2019), although which genomic regions are recombining is not known. Although hypothetically any genomic similarity can trigger recombination, crossovers have strong “preferences”, and will form between whatever chromosomes are present on order of sequence similarity first (Grandont et al. 2014). In the absence of homologous pairing partners, recombination will occur most frequently between the most closely-related (or possibly largest) homoeologous regions (Nicolas et al. 2007, 2009; Mason et al. 2014). Regions of primary and secondary (resulting from ancestral triplication) homoeology have been well-defined for quite some time for the A and C genomes (Parkin et al. 2003; Schranz et al. 2006; Cheng et al. 2013), and to a lesser extent the B genome (Lagercrantz and Lydiate 1996). Now, with the availability of reference genome sequences, these relationships have been even better elucidated (The Brassica rapa Genome Sequencing Project Consortium 2011; Chalhoub et al. 2014; Parkin et al. 2014; Yang et al. 2016; Wang et al. 2019), and subsequently can be used to predict the probable locations of genomic introgressions, even though we are still lacking a lot of information about where crossovers are most likely to form.

Conventionally, we can boost our chances of transferring desirable loci by selecting good targets: single, major gene effects, present in close relatives to our crop of interest. However, several agronomic traits of interest, including yield, plant height and flowering time, are controlled by many genes and heavily influenced by the environment, and thus present a greater challenge. The genetic control or mode of inheritance of a desired trait can be examined through marker analysis or by observing how traits segregate in progenies. Owing to the recent advancement in genotyping, gene editing and marker technologies, the characterization and introgression of our gene of interest, genomic regions (complex traits) or even pyramiding of multiple QTLs can readily be done. In a number of studies, introgression was successfully achieved via a combination of different hybridization schemes. In general, creation of a suitable mapping population to elucidate the genetic control of the trait, followed by association of phenotypes with genotypes to identify genomic regions of interest and subsequent development of marker assisted selection (MAS), is an excellent strategy to facilitate production of introgression lines. For instance, to introgress *Sclerotinia* resistance into rapeseed, Mei et al. (2020) transferred multiple resistant loci from wild *B. oleracea* through backcrossing, selfing and MAS. A different approach was used by Mei et al. (2015) to introgress *Sclerotinia* resistance from *B. incana* (a wild relative of *Brassica oleracea*) *into B. napus* bridged by a hexaploidy step.

Tracking of introgressions can be sped up by marker-assisted selection, or possibly even by genomic selection in the case of multi-locus traits being transferred between close relatives. Marker-assisted selection has been effectively used to track B-genome introgressions related to *Sclerotinia* disease resistance in *Brassica napus* (Navabi et al. 2010), to produce higher-quality restorer lines carrying the *Rfo* restorer gene for the *Ogura* CMS system in *B. juncea* by reducing the size of the radish introgression (Tian et al. 2014), and to map and move clubroot resistance gene *Rpb1* from *B. rapa* into *B. napus* (Chu et al. 2013). Genome-wide marker assisted-selection has also been used in several studies to recover subgenome-substitution or resynthesized lines. This approach has been used to produce “new-type” *B. napus* (AACC) with an A genome from *B. rapa* (A^r^) and a C genome (C^c^) from *B. carinata* (Xiao et al. 2010), through selection for A^r^ and C^c^ alleles over A^n^ and C^n^, as well as to extract the *B. napus* A genome by eliminating the C genome (Pelé et al. 2017). Hence, genome-wide marker-assisted selection may be worth considering in the case of complex traits which are being moved between species which share a subgenome (e.g. species within the Triangle of U).

## Conclusions

In this review, we introduce potential impacts of climate change on crop production and the Brassicaceae crops, provide a reference for useful traits present in each of the *Brassica* “Triangle of U” species and then offer concrete advice for structuring and optimizing introgression breeding programs. Success in transferring agronomically relevant traits between species depends on factors such as similarity between the source (e.g. wild relative) and target (e.g. crop) genomes, the ease of hybrid production, the frequency and distribution of crossovers in the interspecific hybrid meiosis, and subsequently the ease of recovery of introgression lines. Regardless of the considerable difficulties involved in the use of wild relatives for crop improvement, this method offers a great deal of as-yet unexplored potential for the improvement of *Brassica* crops, and in improving crop resilience and resistances in the face of global climate change.
